# Rapid detection of resistance to carbapenems and cephalosporins in *Enterobacteriaceae* using liquid chromatography tandem mass spectrometry

**DOI:** 10.1371/journal.pone.0206842

**Published:** 2018-11-09

**Authors:** Manal Tadros, Lee Goneau, Alexander Romaschin, Michael Jarvis, Larissa Matukas

**Affiliations:** 1 Department of Laboratory Medicine & Pathobiology, University of Toronto, Toronto, Ontario, Canada; 2 Division of Microbiology, St. Michael’s Hospital, Toronto, Ontario, Canada; 3 Department of Microbiology, Public Health Ontario, Toronto, Ontario, Canada; 4 Sciex, Concord, Ontario, Canada; Universidad de Santiago de Compostela, SPAIN

## Abstract

Carbapenemase producing *Enterobacteriaceae* (CPE) are becoming a global healthcare concern. Current laboratory methods for the detection of CPE include screening followed by confirmatory phenotypic and genotypic tests. These processes would generally take ≥72 hours, which could negatively impact patient care and Infection Control practices. To this end, we developed a protocol for rapid resistance testing (RRT) to detect hydrolysis in a panel of beta lactam antibiotics consisting of ampicillin, cefazolin, cefotaxime and imipenem, using liquid chromatography tandem mass spectrometry. Ninety—nine beta lactamase producing *Enterobacteriaceae* isolates were used to evaluate the RRT method, 54 isolates were CPE and 45 isolates were Class A or AmpC beta lactamase producing *Enterobacteriaceae* but not carbapenemase producers. We also tested 10 *E*.*coli* isolates that were susceptible to ampicillin, cefazolin, cefotaxime and imipenem. Receiver Operating Characteristic (ROC) Curves analysis showed that imipenem had a sensitivity and a specificity of 100% for crabapenemase detection at hydrolysis cut off values that are greater than 50% and less than or equal to 80%. The RRT protocol can be conducted in a time frame of less than 2 hours. This preliminary study shows that the rapid resistance testing protocol might have utility for the rapid detection of CPE. Additional work with a greater number and variety of beta- lactamase producing *Enterobacteriaceae* isolates is required to validate these preliminary findings.

## Introduction

Antibiotic resistant organisms such as carbapenemase producing *Enterobacteriaceae* (CPE) and extended spectrum beta lactamase (ESBL) producing *Enterobacteriaceae* have become a worldwide healthcare challenge [1]. In Canada, the rates of (ESBL)-producing *Escherichia coli* and *Klebsiella pneumoniae* have increased steadily over the last decade [2]. A more worrisome fact is that the overall CPE rates have increased in Canada by approximately 33% from 2010 (0.06 per 1,000 patient admissions) to 2014 (0.08 per 1,000 patient admissions) [3]. Timely laboratory detection of these organisms assists with appropriate antibiotic therapy as well as rapid implementation of infection control measures to halt nosocomial transmission of these organisms [4]. Current laboratory methods for detection of CPE include screening followed by confirmatory phenotypic and genotypic tests. These processes would generally take ≥72 hours [5–9]. To expedite this process, mass spectrometry has been utilized to detect hydrolysis of selected beta-lactam antibiotics such as ertapenem and meropenem [10–12]. In addition, bottom-up proteomics has been utilized to detect peptides specific to ESBL enzymes such as CTX-M [13]. The purpose of our study was to evaluate if a protocol for rapid resistance testing (RRT) using a panel of beta-lactam antibiotics and liquid chromatography tandem mass spectrometry (LC-MS/MS) could accurately identify CPE and distinguish their pattern of hydrolysis from other beta lactamases namely, Class A and AmpC enzymes.

## Materials and methods

### Selection of bacterial isolates

Fifety-four previously characterized *Enterobacteriaceae* isolates carrying the 3 CPE genes that are most commonly encountered in Toronto were selected for testing: 22 isolates were producers of New Delhi metallo beta lactamase enzyme (NDM), 21 isolates were *Klebsiella pneumoniae* carbapenemase producers (KPC) and 11 isolates were producers of Oxacillinase 48 or 48-like enzymes (OXA-48).

In addition, forty-five beta lactamase producing isolates (32 Class A ESBL and 13 AmpC) were included in our study to test the specificity of the Rapid Resistance Testing (RRT) method for CPE detection and 10 *E*.*coli* isolates that were susceptible to ampicillin, cefazolin, cefotaxime and imipenem were included as negative controls.

The isolates were a representative sample of the significant *Enterobacteriaceae* genera and species isolated at St. Michael’s Hospital, Toronto, Canada clinical microbiology laboratory (e.g. *K*. *pneumoniae*, *E*.*coli*, *Enterobacter* spp. and others). The majority of CPE isolates were provided by our reference laboratory, Public Health Ontario Laboratory (PHOL).

### Conventional methods for the detection of carbapenemase enzymes in *Enterobacteriaceae*

Our microbiology laboratory screened all *Enterobacteriacae* isolates for reduced susceptibility such that those with a zone of inhibition diameter of <25 mm for meropenem and/or ertapenem, undergo further testing for carbapenemase production. Phenotypic confirmation of carbapenemase production was performed at our laboratory using the KPC and MBL Confirm Kit (Rosco Diagnostica) with a temocillin 30μg disk to facilitate OXA-48 detection [14]. Final confirmation of CPE was determined by molecular detection of carbapenemase genes performed at the reference laboratory, The Public Health Ontario Laboratory using a laboratory developed multiplex real time PCR assay to detect *bla*_KPC_, *bla*_NDM_, *bla*_OXA-48-like_, *bla*_GES_, *bla*_VIM_, and *bla*_IMP_ as described previously [9]

### Conventional methods for the detection of Class A and AmpC ESBL enzymes in *Enterobacteriaceae*

*Enterobacteriaceae* isolates other than *P*.*mirabilis* were flagged as potential ESBL producers by Vitek2 system (bioMérieux, Marcy l’Etoile, France) if they had a minimal inhibitory concentration (MIC) of ≥8 μg/ml for cefpodoxime and/or an MIC of ≥2 μg/ml for cefotaxime or ceftazidime [15]. *P*.*mirabilis* isolates that had an MIC of ≥ 2 μg/ml for cefpodoxime, cefotaxime or ceftazidime were flagged as potential ESBL producers [15]. These isolates were further evaluated for the presence of ESBL by the double disk diffusion test (DDD) which is considered the gold standard method for detection of ESBL at our laboratory [7,8]. The DDD was performed according to the Institute for Quality Management in Healthcare (IQMH) [7] to confirm ESBL production and differentiate Class A ESBL organisms from AmpC producers. Briefly, a suspension of the test organism equivalent to a 0.5 McFarland turbidity standard was prepared and inoculated on a Mueller-Hinton agar plate. An amoxicillin/clavulanic acid disk (20/10 μg) was placed in the centre of the inoculated plate. Disks of ceftazidime (30 μg), ceftriaxone (30 μg), cefpodoxime (10 μg) and aztreonam (30 μg) were placed 15–20 mm (edge-to-edge) from the amoxicillin/ clavulanic acid disk. A cefoxitin (30 μg) disk was placed in the available space remaining on the plate. After overnight incubation the zones for signs of beta- lactamase activity were observed in the area where the amoxicillin/clavulanic acid and cephalosporin or aztreonam diffusion zones overlap. Beta- lactamase inactivation was evidenced by enhanced killing (i.e. enlarged zone of inhibition) of the organism in the area of the drug combination compared to the drug alone [7].

### Incubation and sample extraction for mass spectrometry

A detailed version of the protocol for rapid resistance testing has been submitted to Protocols.io.

A sterile 1μl loop was used to suspend a fresh (18–24 hours old), pure colony of each isolate, at concentrations of 0.5 to 1.0 McFarland, in 2 milliliters of Mueller-Hinton broth containing a mixture of ampicillin, cefazolin, cefotaxime and imipenem all at individual concentrations of 0.5 μg/ml. The rationale for using a low concentration of the antibiotics in the quadrupole mixture was to minimize antibiotic competition for enzyme. The suspensions were incubated for one hour at 37°C with gentle agitation at 200 cycles per minute in a shaking incubator. The mixture of the four antibiotics incubated with bacterial isolates was then tested using positive electrospray ionization following a rapid extraction protocol and HPLC separation. Following the incubation, antibiotics were extracted by adding 600 microliters of methanol to 300 microliters of the incubation mixture in 2 ml Eppendorf tubes. To ensure that the results were reproducible, two samples from each class of resistant organism were run in triplicate on separate days. To exclude the possibility of auto hydrolysis, a control strain (*E*.*coli* ATCC 25922) susceptible to all antibiotics was included with multiple runs. In addition, 10 *E*.*coli* isolates cultured from clinical specimens, susceptible to the 4 antibiotics by the Vitek2 system were included as negative controls.

#### Internal standard

Prior to extraction with methanol ten microliters of oxazepam (5 μg/ml in methanol) was added to the bacterial incubation mixture as an internal standard with subsequent vortexing for five seconds. Oxazepam was used as a surrogate for antibiotic internal standards to correct for variations in antibiotic recovery and LC-MS/MS conditions.

### LC-MS/MS for the detection of hydrolysis of beta-lactam antibiotics

A triple quadrupole mass spectrometer was mated to a liquid chromatography system to facilitate molecular separation. Following methanol extraction, the antibiotic- organism mixtures were then centrifuged at 35,000x g in a mini-centrifuge for five minutes to remove precipitated protein. Six hundred microliters of the supernatant was then removed into 2 ml glass injection vials and mixed with 300 microliters of distilled water. This extract was capped and subjected to liquid chromatography tandem mass spectrometry. LC-MS/MS was performed on a 3200 Qtrap mass spectrometer (Sciex Concord ON, Shimadzu LC20 Integrated HPLC system, Guelph ON). The antibiotics of interest were first separated using a C-8 reverse phase chromatography column which separated the compounds based on the order of decreasing polarity with subsequent infusion into an electrospray mass spectrometer source operating in positive mode.

Multiple Reaction Monitoring (MRM) was used to quantify hydrolysis and improve specificity. In MRM, the ions of interest (ampicillin, cefazolin, cefotaxime and imipenem) were preselected with the mass filter in the first quadrupole (Q1). The selected ion specific for the target antibiotic then entered the second quadrupole (Q2) where it underwent collision-fragmentation. The daughter ion specific to the parent chosen in Q1 was subsequently mass filtered in the third quadrupole (Q3). The specificity of the assay was based on the retention time in the liquid chromatography phase and the subsequent specific parent to daughter ion transition (MRM). Results were reported as percent hydrolysis based on loss of parent drug. Appearance of hydrolyzed ampicillin product +18 atomic mass units (amu) was used as an additional confirmation of ampicillin hydrolysis. The hydrolysis products of the three other antibiotics were not visualized likely due to instability of the molecules or low product yield in positive ionization mode.

### Receiver operating characteristic (ROC) curves analysis

ROC curves were constructed by calculating sensitivities and specificities at various thresholds for cefotaxime and imipenem. Confidence intervals were estimated with bootstraps and optimal thresholds were selected based on highest sum of sensitivity and specificity. All calculations and plots were done in R 3.5 using package “pROC”

## Results

**Fig 1:**
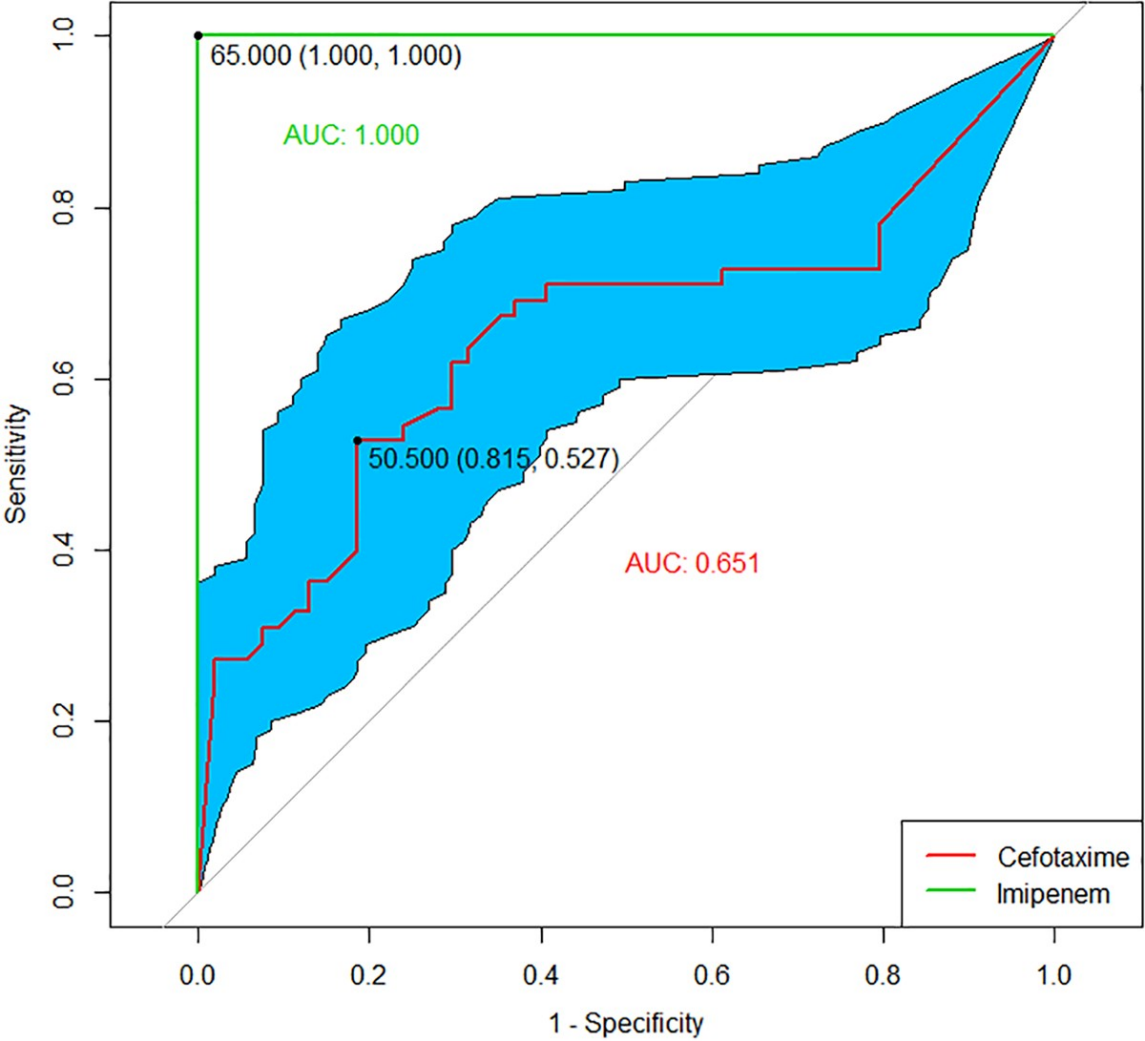
ROC analysis of imipenem (green) and cefotaxime (red). AUC: area under the curve.

A summary of the tested organisms and their beta-lactamase profiles is provided in Table 1. The results of RRT by LC-MS/MS analysis are summarized in Table 2. ROC curves showed that a cut off for imipenem hydrolysis that is greater than 50% and less than or equal to 80% would have 100% sensitivity and 100% specificity for CPE detection with an area under the curve (AUC) = 1. Cefotaxime showed poor performance for CPE detection with an AUC = 0.65 (Fig 1).

**Table 1:** Characterization of *Enterobacteriaceae* isolates.

	Number of isolates expressing the indicated beta-lactamase enzyme						
Genus and species	OXA 48-like	KPC	NDM	Class A ESBL	AmpC ESBL	No beta-lactamase	Total
*E*.*coli*	0	5	12	23	6	10	56
*Klebsiella pneumoniae*	10	9	7	8	0	0	34
*Enterobacter cloacae* complex	0	2	1	1	3	0	7
*Citrobacter freundii*	0	2	0	0	1	0	3
*Serratia marcescens*	0	1	0	0	1	0	2
Other	1	2	2	0	2	0	7
Total	11	21	22	32	13	10	109

**Table 2:** Percent hydrolysis of antibiotics in isolates expressing different beta-lactamases by the RRT method.

	Rapid Resistance Testing (RRT) results; average percent hydrolysis;(range)			
Beta-lactamase enzyme type/Number of isolates (n)	Ampicillin	Cefazolin	Cefotaxime	Imipenem
OXA 48-like (11)	98 (89–100)	78(61–100)	73(16–100)	100(NA)
KPC (21)	96(85–100)	75(65–100)	65(0–100)	99.5(95–100)
NDM (22)	87(40–100)	76(35–100)	83(25–100)	95(80–100)
Class A ESBL (32)	89.8(34–100)	96.4(60–100)	75.5(0–100)	0(NA)
Class C ESBL (13)	60.9(20–100)	86.4(20–100)	30(0–100)	11(0–50)
No phenotypic evidence of beta-lactamases (10)	0 (NA)	0 (NA)	0 (NA)	0 (NA)

NA; not applicable

**Fig 2:**
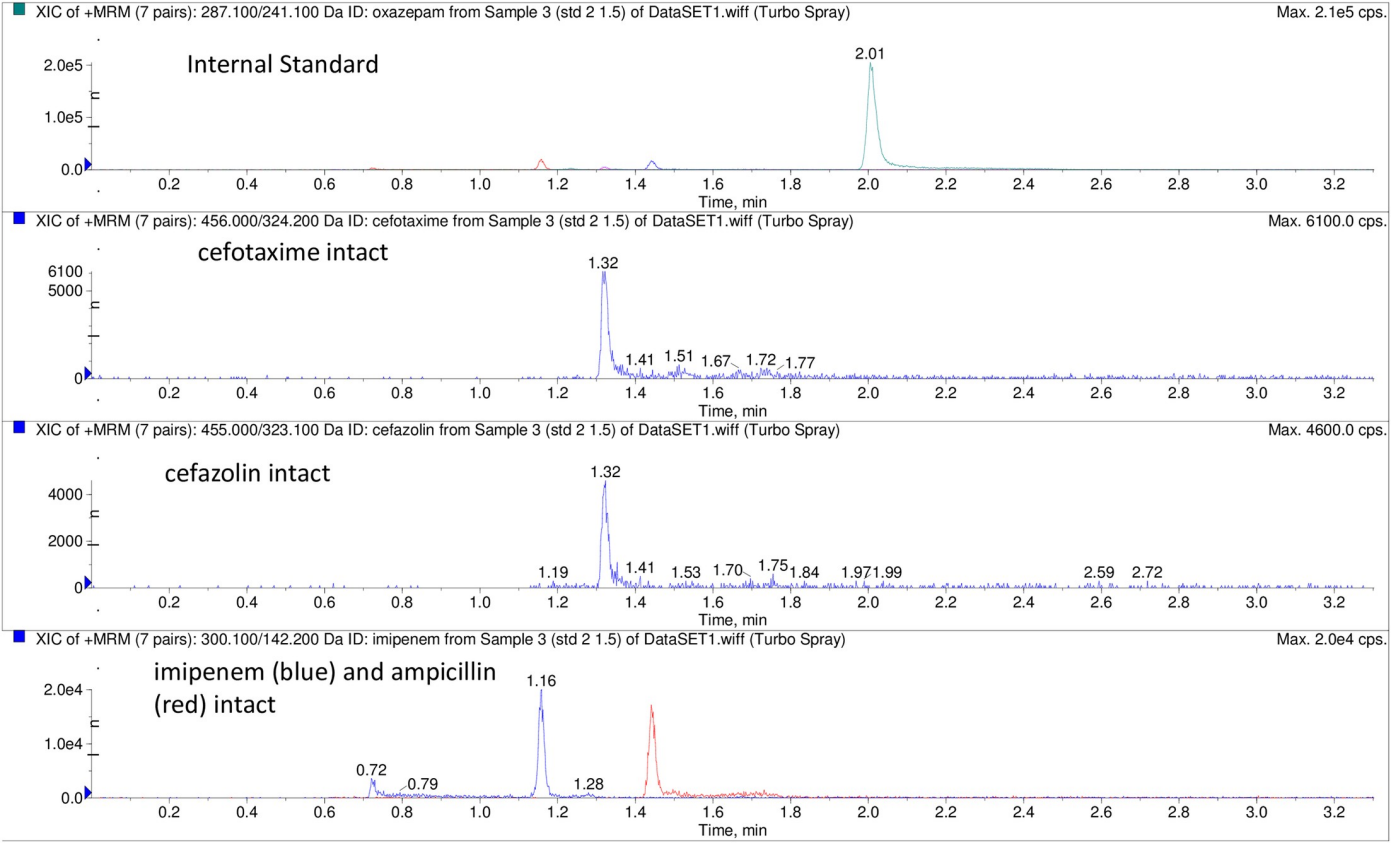
MRM (multiple reaction monitoring) chromatogram showing intact profiles of cefazolin, cefotaxime, imipenem and ampicillin respectively. The intact profiles were demonstrated after incubation of the antibiotic mixture with *E*.*coli* ATCC 25922 (sample 3). The profile of the internal standard (oxazepam) is shown above the profiles of the intact antibiotic panel.

**Fig 3:**
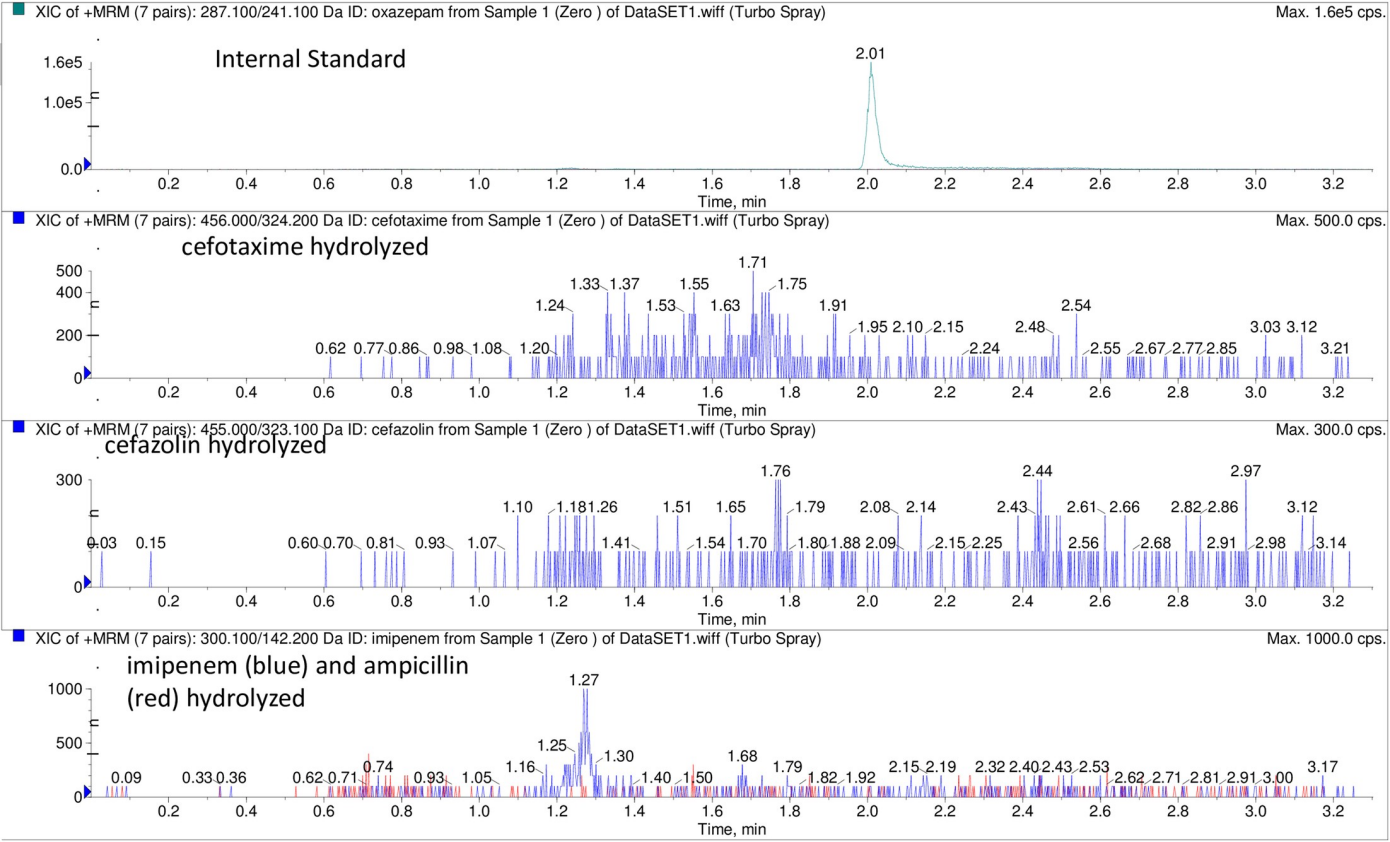
MRM (multiple reaction monitoring) chromatogram showing the profiles of the remaining amounts of cefazolin, cefotaxime, imipenem and ampicillin respectively after hydrolysis. The hydrolyzed profiles were demonstrated after incubation with a CPE isolate (sample 1). The profile of the internal standard (oxazepam) is shown above the profiles of the hydrolyzed antibiotic panel.

The profile of intact and hydrolyzed antibiotic molecules is illustrated in Figs 2 and 3 respectively. The intact profiles were demonstrated when the tested antibiotics were placed in broth free of bacteria as well as when they were incubated with a control susceptible strain (*E*.*coli* ATCC 25922).

All CPE isolates (n = 54) that were tested showed substantial hydrolysis of imipenem to 80% or greater following the incubation cycle, An NDM producing *Morganella morganii* isolate hydrolysed imipenem to 80% of the original value after 1 hour of incubation. Four AmpC isolates were able to hydrolyze imipenem but not in excess of 50% of the original dose. Applying ROC analysis, cut offs of imipenem hydrolysis of >50% and ≤80% had 100% sensitivity and specificity.

Interestingly, 10 CPE isolates, showed weak hydrolysis (below 50%) of cefotaxime. Applying ROC analysis, cefotaxime had an AUC of 0.65 for CPE detection, suggesting that cefotaxime hydrolysis is a poor predictor of CPE. None of the Class A ESBL isolates (n = 32) hydrolyzed imipenem. A control strain (*E*.*coli* ATCC 25922) susceptible to all antibiotics showed no detectable hydrolysis of any of the antibiotics tested (Fig 1) and was repeated in triplicate with the same results. (data not shown). Ten *E*.*coli* isolates that tested susceptible to ampicillin, cefazolin, cefotaxime and imipenem by conventional methods, showed no hydrolysis of any of the 4 antibiotics when tested by the RRT protocol.

## Discussion

The Clinical Laboratory Standards Institute (CLSI), defines the breakpoints for non-susceptibility of *Enterobacteriaceae* to ertapenem and meropenem as a zone of inhibition of <22 mm and <23 mm respectively [15,16]. However, our microbiology laboratory screened all *Enterobacteriacae* isolates for reduced susceptibility such that those with a zone of inhibition diameter of <25 mm for meropenem and/or ertapenem, undergo further testing for carbapenemase production. This is in accordance with The European Committee on Antimicrobial Susceptibility Testing (EUCAST) screening cut-off values [17] and the Institute for Quality Management in Healthcare (IQMH) [7] as well as other studies by Dortet et al [18] and Fattouh et al [19].

Recently, many microbiology laboratories have implemented protocols for the rapid identification of organisms from significant cultures using MALDI-TOF MS [20,21]. This success in rapid identification was unfortunately not paralleled by an equal success in developing novel methodologies for rapid susceptibility testing. Several studies have described the use of MS instruments to detect beta-lactamase activity [22–27]. Most of these studies have shown that mass spectrometry is a promising tool for detection of beta-lactamase expression. Our study showed the proposed RRT protocol is a highly sensitive method for the rapid detection of CPE. All CPE isolates included in this study were able to hydrolyze imipenem to extents ≥80% under the given conditions. In our study, we found that not all CPE are potent hydrolyzers of cefotaxime, 10 of 54 CPE isolates tested produced weak hydrolysis of cefotaxime (below 50%). This finding has been also reported by Hrabák and co-workers [28], especially in OXA-48-like enzyme producers that show *in vitro* susceptibility to cephalosporins, an issue which further complicates their detection by conventional phenotypic methods. Although establishing the clinical impact and therapeutic implications of carbapenemase producers that weakly hydrolyze cephalosporins is beyond the scope of our study, this could be a subject of significant interest in future studies.

The observation that some AmpC enzyme producers can hydrolyze carbapenems has been previously described [28]. Jacoby noted the role played by a decrease in the number of porin channels in increasing the efficiency of AmpC enzymes in some organisms. This is due to the entrapment of the antibiotic in the periplasmic space making it more accessible for hydrolysis by AmpC enzymes [29]. Cefepime might help in discriminating AmpC producers that are able to partially hydrolyze imipenem, in the setting of porin changes, from true carbapenemase producers as cefepime resists hydrolysis by AmpC enzymes to a large extent [29]. An additional measure would be shortening the incubation time of the organism with the tested antibiotics. This would likely decrease the ability of AmpC enzymes to hydrolyze imipenem. Previous studies have utilized MALDI-TOF protocols for testing susceptibility to individual antibiotics in non-multiplexed mixtures at much higher concentrations of bacteria and antibiotics to compensate for sensitivity issues [22,27]. A known limitation to the use of MALDI-TOF systems for detection of beta-lactam resistance has been the need to calibrate instruments at lower mass ranges for this analysis and the need to use a different matrix from the one commonly used for bacterial identification [11]. The multiplex panel of antibiotics used in our study, allowed us to examine hydrolysis patterns of different carbapenemase producing *Enterobacteriaceae* when tested against multiple beta lactam agents. In addition, the successful hydrolysis of multiple antibiotics within the same reaction reflects the possibility of expanding the panel of antibiotics to make it useful in detecting other beta-lactamases and differentiating Class A from AmpC enzymes. Our study had some limitations; one being the lack of molecular characterization of the class A and AmpC encoding genes. Another limitation is that it is a retrospective study in which archived isolates were used, hence the clinical utility and the impact of providing real time results of RRT on patient care was not evaluated. The RRT protocol can be conducted in a time frame of less than 2 hours from the point of bacterial inoculation into the reaction broth which could substantially reduce the time required to identify carbapenemase producing *Enterobacteriaceae*.

## Conclusions

This preliminary study shows that the RRT protocol might have utility as a quick tool for detection of CPE It can be possibly modified to extend its use to be able to detect most beta lactamases in *Enterobacteriaceae* using a single panel of beta lactam antibiotics. Additional studies are needed to validate the results produced by this preliminary work and examine its clinical utility.
